# Collagen Fiber Maturity and Architecture in MVP-Associated Fibrosis Quantified by Digital Pathology

**DOI:** 10.3390/cells14191536

**Published:** 2025-09-30

**Authors:** Ranan Phookan, Jordan E. Morningstar, Brian Loizzi, Antonia Van Kampen, Cortney Gensemer, Maja-Theresa Dieterlen, Ricardo Spampinato, Louis Petitjean, Mathieu Petitjean, Taylor Petrucci, Roman Fenner, Jake Griner, Kathryn Byerly, Robert A. Levine, Michael A. Borger, Russell A. Norris

**Affiliations:** 1 Department of Regenerative Medicine and Cell Biology, College of Medicine, Medical University of South Carolina, Charleston, SC 29407, USA; phookan@musc.edu (R.P.); morningj@musc.edu (J.E.M.); loizzi@musc.edu (B.L.); gensemer@musc.edu (C.G.); petrucct@musc.edu (T.P.); fennerro@musc.edu (R.F.); griner@musc.edu (J.G.); byerlyk@musc.edu (K.B.); 2 Cardiac Ultrasound Laboratory, Massachusetts General Hospital, Harvard Medical School, Boston, MA 02114, USA; antoniavankampen@gmail.com (A.V.K.); levine.robert@mgh.harvard.edu (R.A.L.); 3 University Department of Cardiac Surgery, Leipzig Heart Center, Struempellstrasse 39, 04289 Leipzig, Germany; maja-theresa.dieterlen@helios-gesundheit.de (M.-T.D.); spampinatoricardo@gmail.com (R.S.); michael.borger@helios-gesundheit.de (M.A.B.); 4 PharmaNest Inc., Princeton, NJ 08540, USA; louis.petitjean@pharmanest.com (L.P.); mathieu.petitjean@pharmanest.com (M.P.)

**Keywords:** mitral valve prolapse, cardiac fibrosis, digital pathology, collagen, mechanoregulation

## Abstract

Recent evidence demonstrates that mitral valve prolapse (MVP) increases mechanical stress on the subvalvular apparatus and is linked to regional myocardial fibrosis and life-threatening ventricular arrhythmias. However, current surgical guidelines do not account for the extent of myocardial fibrosis or the severity of leaflet involvement, both key features of arrhythmogenic MVP. To address this gap, we conducted histopathological analysis of endomyocardial biopsies from patients with MVP and regionalized myocardial fibrosis (n = 6) who underwent mitral valve repair. Using digital pathology-based quantitative image analysis (QIA), we found that fibrosis in peri-papillary biopsies exhibited a significantly higher Morphometric Composite Score compared with remote biopsies (5.68 ± 0.69 vs. 3.71 ± 0.49, *p* = 0.042), reflecting larger, more branched, and more assembled collagen fibers, indicative of a mature and persistent fibrotic phenotype. These findings suggest that myocardial scarring in MVP is well-established by the time of surgery and underscore the potential value of earlier surgical intervention to reduce the risk of arrhythmia and preserve post-operative left ventricular function.

## 1. Introduction

Mitral valve prolapse (MVP) is a common valvular disorder affecting 2–3% of the general population and is defined by the systolic displacement of one or both mitral valve leaflets into the left atrium due to excess leaflet tissue or elongated chordae tendineae [1,2]. While many patients remain asymptomatic, MVP can progress to mitral regurgitation (MR), wherein improper coaptation of the leaflets during systole results in retrograde blood flow into the left atrium. MR can lead to downstream complications such as left atrial enlargement, atrial fibrillation, pulmonary hypertension, and congestive heart failure, conditions that frequently serve as the primary indications for surgical mitral valve repair or replacement [3,4].

Emerging clinical and translational research has highlighted that the pathophysiology of MVP extends beyond MR-driven volume overload. A growing body of evidence supports a direct role of leaflet prolapse in the development of ventricular arrhythmias, sudden cardiac death (SCD), and myocardial fibrosis, independent of MR severity [3,5,6,7,8,9,10,11,12]. Myocardial fibrosis has been identified in up to one-third of MVP patients, including those without significant MR, and has been implicated in arrhythmic risk stratification [13]. Moreover, in matched patient cohorts, those with MVP exhibit a higher prevalence and regional burden of fibrosis compared to patients with MR from other etiologies. Notably, cases of SCD in MVP are often associated with bileaflet prolapse, mid-systolic clicks, and T-wave inversions, features characteristic of arrhythmogenic MVP, even when MR is only moderate [14,15,16,17].

The prevailing hypothesis is that abnormal mechanical stress imposed by the prolapsing leaflet contributes to myocardial remodeling, particularly in the subvalvular apparatus. Histopathological and cardiac MRI studies have localized MVP-associated fibrosis to the papillary muscles, inferobasal left ventricular wall, and mid-myocardial layers [7,8,11,18]. Our group previously reported regionalized myocardial fibrosis in both human MVP patients and a genetic murine model of nonsyndromic MVP, with lesions concentrated adjacent to the posterior papillary muscle and inferobasal myocardium [8]. These findings were further supported by ex vivo bioreactor models, which demonstrated that leaflet prolapse, especially bileaflet prolapse, exacerbates mechanical strain on the chordae tendineae and subvalvular myocardium [5,19]. Collectively, these data suggest that prolapse-induced mechanical stress initiates fibrotic remodeling in localized myocardial regions, independent of MR-induced volume overload.

Despite this growing recognition of fibrotic remodeling in MVP, several key questions remain unresolved. Most notably, it is unclear whether fibrosis observed at the time of surgical intervention represents a static, irreversible process or a potentially dynamic and reversible one. Post-surgical imaging studies have provided conflicting results regarding the regression of myocardial fibrosis following valve repair. Moreover, histologic characterization of myocardial fibrosis in MVP remains limited, particularly at the level of collagen fiber architecture, maturity, and organization. These features could help distinguish between reversible and irreversible remodeling.

Advances in digital pathology are now enabling high-resolution, unbiased quantitative assessment of tissue fibrosis. This emerging field combines whole-slide imaging with computational analysis to evaluate tissue morphology, architecture, and extracellular matrix composition. One such tool, the FibroNest platform, utilizes quantitative image analysis (QIA) algorithms to extract fibrosis parameters from digitized tissue slides, including collagen fiber length, thickness, branching, reticulation, and assembly. FibroNest has been successfully deployed in clinical trials evaluating antifibrotic therapies in patients with metabolic dysfunction-associated steatohepatitis (MASH), where its ability to distinguish between mature and immature collagen architecture provides insight into fibrosis progression and treatment response [20,21,22,23,24,25].

Given its ability to characterize fiber-level collagen remodeling, we applied the FibroNest platform to endomyocardial biopsy samples from MVP patients with regionalized fibrosis undergoing surgical repair. Our aim was to evaluate the histological composition and maturity of MVP-associated myocardial fibrosis using QIA-driven digital pathology. By determining whether the observed fibrosis reflects a persistent, organized matrix or a more immature, potentially modifiable state, we seek to inform the timing of surgical intervention and potentially improve risk stratification for arrhythmogenic complications in MVP.

## 2. Materials and Methods

### 2.1. Study Design and Patient Recruitment

Patients included in this study were prospectively recruited as previously described by our group [8]. All study procedures involving human participants adhered to the ethical standards of the institutional and national research committees and conformed to the Declaration of Helsinki (1974, revised in 2013) and its subsequent amendments. The study protocol was approved by the local ethics committee (protocol number 450/18-ek, 26 June 2019), and written informed consent was obtained from all participants. Six patients undergoing mitral valve repair for severe MR secondary to MVP were enrolled. Exclusion criteria included any comorbid cardiac conditions that could independently cause myocardial fibrosis, such as coronary artery disease, non-valvular cardiomyopathies, aortic stenosis, or a history of prior cardiac surgery. Baseline demographic, comorbidity, and echocardiographic data were collected and summarized for all study participants and previously reported [8].

### 2.2. Tissue Acquisition

During surgical mitral valve repair, two endomyocardial biopsies were obtained per patient. One biopsy was consistently sampled from the inferobasal left ventricular myocardium adjacent to the posteromedial papillary muscle (n = 6). The second biopsy was taken from a remote region of the left ventricle, either the interventricular septum (n = 3) or the apex (n = 3). Specimens were harvested using a scalpel and weighed immediately after collection, with an average sample weight of 84 ± 71 mg. All samples were processed for downstream histopathological and digital analysis.

### 2.3. Histologic Processing, Staining, and Digital Pathology

Tissue specimens were fixed in 10% neutral-buffered formalin for 24 h and processed through graded ethanol and toluene before paraffin embedding. Serial 5 μm sections were prepared using a rotary microtome and mounted on glass slides. Masson’s Trichrome staining was performed using standard protocols. Briefly, slides were mordanted in Bouin’s solution and stained sequentially with Weigert’s hematoxylin, Biebrich scarlet–acid fuchsin, phosphotungstic/phosphomolybdic acid, Aniline Blue, and 1% acetic acid, with deionized water washes between steps. Slides were dehydrated through ethanol and xylene and coverslipped with Cytoseal (Electron Microscopy Sciences, Inc., Hatfield, PA, USA). Whole-slide images were acquired at 40× magnification using a Hamamatsu NanoZoomer digital scanner for downstream computational analysis.

### 2.4. Quantitative Fibrosis Profiling

All samples were analyzed using the FibroNest™ platform (PharmaNest Inc., Princeton, NJ, USA), a cloud-based digital pathology tool that employs quantitative image analysis for high-content fibrosis phenotyping (Figure S1). FibroNest automatically extracted >300 quantitative fibrosis traits (qFTs), spanning collagen content, fiber morphology, and architectural features. Traits included collagen area, density, nodularity, branching complexity, fiber length, thickness, orientation, and higher-order descriptors of heterogeneity and directionality.

The qFTs that exhibited a significant change between the peri-papillary and remote groups (group mean change > 20%, *p* > 0.05) were normalized and integrated into four equi-weighted composite fibrosis scores: Phenotype Fibrosis Composite Score (Ph-FCS), Collagen Composite Score (CCS), Morphometric Composite Score (MCS), and Architecture Composite Score (ACS) (Table S1). These metrics were used for comparative analysis across peri-papillary versus remote regions and across tissue compartments (whole biopsy, myocardium, endocardium). A visual workflow of the FibroNest method and the full list and nomenclature of the 336 fibrosis qFTs have been previously described [20,21]. 

### 2.5. Fiber Segmentation and Classification

To resolve collagen organization at the single-fiber level, images were further analyzed using FiberID, a computational image segmentation pipeline that automatically detects, segments, and parameterizes individual collagen fibers. FiberID measures structural properties such as length, continuity, thickness, and alignment. Beyond detection, FiberID also assigns fibers to structural classes such as fine fibers and assembled fibers. Fine fibers correspond to thin, immature, or loosely organized fibrils, whereas assembled fibers correspond to thick, bundled, mature collagen networks with >30 nodes/branch points. This classification was applied to peri-papillary and remote myocardial regions, enabling evaluation of localized matrix remodeling and differences in fiber class distribution.

### 2.6. Fibrillar Entropy Mapping

To assess collagen network order and heterogeneity, fibrillar entropy mapping was applied. This computational approach quantifies the degree of disorder within fibrillar networks by applying Grey Level Co-occurrence Matrix texture analysis as previously described [25]. This approach generates an additional set of qFTs, that can also be visualized in processed overlays: high entropy values (red/yellow regions) reflect disorganized, heterogeneous collagen deposition, whereas low entropy values (blue/green regions) indicate ordered fibrillar alignment.

### 2.7. Region of Interest (ROI) Annotations

Whole-slide images were subdivided into endocardial and myocardial regions of interest (ROIs) based on histologic landmarks. Endocardium was defined by endothelial lining and subendothelial collagen, while myocardium was delineated by myocyte density and absence of endocardial structures. This compartmentalization allowed layer-specific analyses, including fibrosis quantification across myocardial and endocardial ROIs (Figure S2).

### 2.8. Analytical Performance

The analytical performance of the Ph-FCS was not directly evaluated in this study. However, we hypothesize that it would be comparable to the analytical performance of the FibroNest composite score, whose performance in quantifying fibrosis severity from liver biopsies of patients with MASH has been extensively reported. Specifically, the Ph-FCS index demonstrated a coefficient of variability between 0.21% and 0.29% (<0.3%) for repeatability (mid- to high-fibrosis severity, N = 45), 3.33% to 5.95% for reproducibility (N = 45), and a mean (median) coefficient of variability of 4.5% (4.0%) for histological variability due to sectioning and staining (N = 45) [20,21].

### 2.9. Statistical Analysis

All quantitative data are presented as mean ± standard deviation unless otherwise specified. Differences in composite scores between groups (e.g., papillary vs. remote,) were assessed using unpaired Mann–Whitney U tests due to the non-parametric distribution of fibrosis traits. A *p*-value < 0.05 was considered statistically significant. Graphs were generated using GraphPad Prism 10 and are presented as box-and-whisker plots with means denoted by center lines, boxes representing interquartile ranges, and whiskers extending to minimum and maximum values.

## 3. Results

To characterize the fibrotic microarchitecture associated with mitral valve prolapse (MVP), we performed histologic and QIA-based digital pathology analyses on paired endomyocardial biopsies from six patients undergoing surgical mitral valve repair. Biopsies were obtained from the inferobasal myocardium adjacent to the papillary muscle (“peri-papillary”) and from a remote intraventricular region (apex or septum) serving as a within-patient control.

### 3.1. Histological Assessment Confirms Regional Fibrosis in MVP

**Figure 1:**
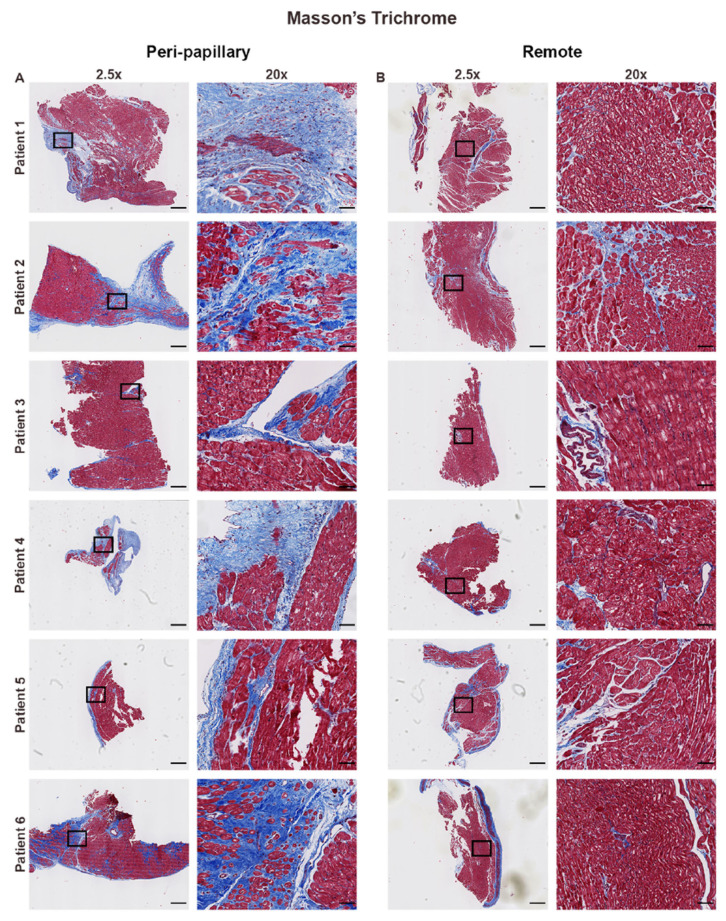
Regional differences in collagen deposition by Masson’s trichrome staining. Representative Masson’s trichrome stained endomyocardial biopsies from six patients with mitral valve prolapse (MVP). (**A**) Peri-papillary region biopsies show extensive fibrosis, including interstitial and replacement fibrosis, with marked collagen deposition (blue) interspersed among cardiomyocytes (red). High magnification (20×) highlights dense and disorganized extracellular matrix expansion adjacent to the papillary muscle. (**B**) Remote biopsies, obtained from the left ventricular apex or interventricular septum, display preserved myocardial architecture with minimal interstitial fibrosis and sparse collagen accumulation. Scale bars represent 600 µm (2.5×) and 108 µm (20×).

Masson’s trichrome staining revealed striking regional differences in collagen distribution (Figure 1). Peri-papillary samples displayed extensive endocardial thickening with both replacement and interstitial fibrosis surrounding cardiomyocytes, whereas remote biopsies largely retained preserved myocardial architecture with minimal fibrosis. To move beyond qualitative assessment, we next applied computational tools to quantify and classify collagen architecture across regions.

### 3.2. Collagen Fiber Architecture Demonstrates Greater Maturity and Complexity in Papillary Region

Quantitative analysis using the FibroNest platform extracted over 300 fibrosis traits, which were summarized into composite scores for phenotypic fibrosis, collagen content, fibrosis architecture, and morphometrics (Table S1). While these measures highlighted broad differences, we sought to interrogate the microstructural features of collagen fibers more directly. For this purpose, we employed FiberID, an automated segmentation tool that identifies, quantifies, and classifies individual fibers within tissue sections.

**Figure 2:**
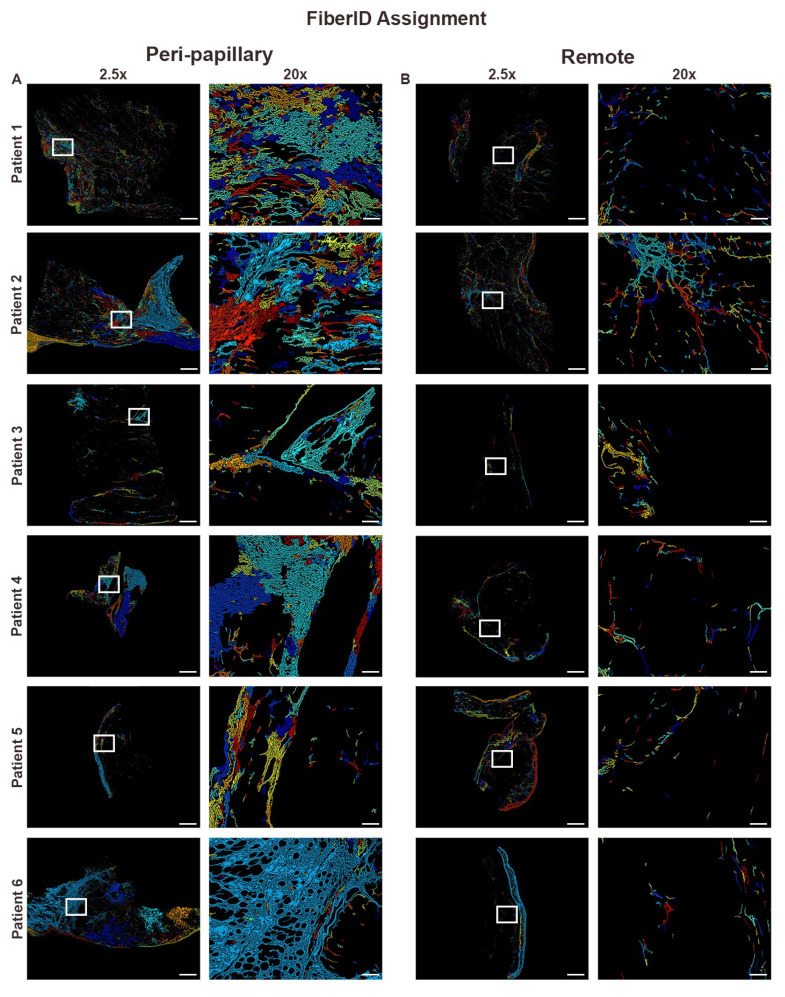
FiberID-based computational analysis of collagen fiber architecture. FiberID analysis of paired biopsies from six MVP patients. (**A**) Papillary region biopsies exhibit dense networks of aligned and continuous collagen fibers, with prominent representation of thick bundled fibers (cyan, blue, and red assignments). High magnification views (20×) reveal organized and tightly packed bundles, reflecting extracellular matrix remodeling. (**B**) Remote biopsies demonstrate sparse, discontinuous, and less aligned collagen fibers, with lower density and reduced organization. Scale bars represent 600 µm (2.5×) and 108 µm (20×).

**Figure 3:**
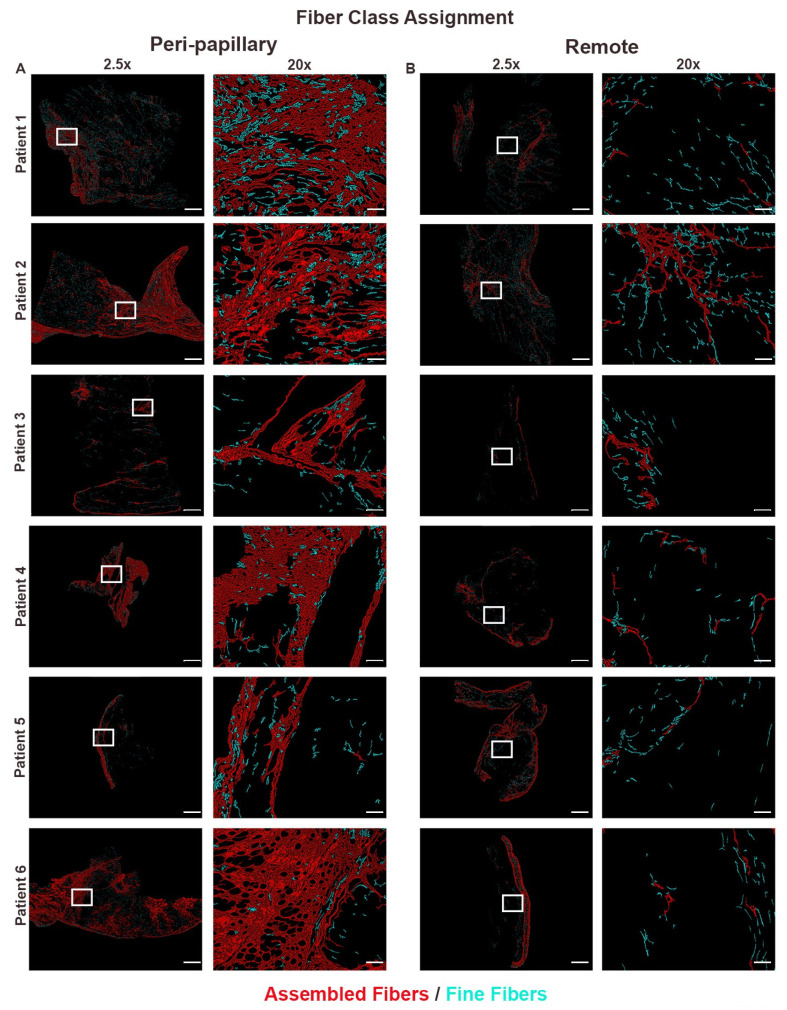
Fiber class assignment highlights regional shifts toward assembled collagen fibers. Fiber class assignment applied to paired biopsies from six MVP patients. (**A**) Papillary regions are enriched in assembled fibers (red), forming dense, interconnected collagen networks consistent with mature fibrosis, with relatively few fine fibers (cyan). (**B**) Remote regions contain predominantly fine fibers with minimal assembled fiber accumulation, reflecting preserved extracellular matrix architecture. Scale bars represent 600 µm (2.5×) and 108 µm (20×).

**Figure 4:**
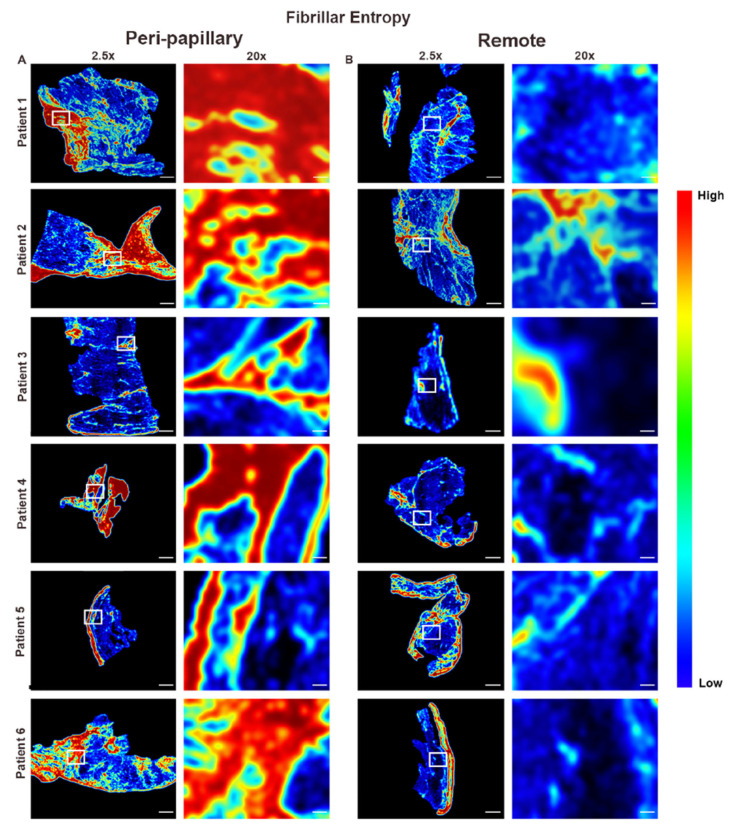
Fibrillar entropy mapping reveals localized disorganization in papillary regions. Heatmap representations of fibrillar entropy applied to peri-papillary (**A**) and remote (**B**) biopsies from six MVP patients. Peri-papillary regions demonstrate elevated entropy, with heterogeneous areas of high disorder (red/yellow), indicating disrupted and complex collagen fiber organization. In contrast, remote regions show consistently low entropy (blue/green), reflecting ordered and stable fibrillar architecture. Scale bars represent 600 µm (2.5×) and 108 µm (20×).

FiberID analysis demonstrated that peri-papillary regions contained dense, aligned, and continuous collagen networks, in contrast to the sparse, fragmented, and disorganized fibers observed in remote myocardium (Figure 2). To further refine these findings, we examined fiber class distribution, which distinguishes thick, bundled assembled fibers from thinner fine fibers. Fiber class assignment revealed that peri-papillary tissue was dominated by assembled fibers consistent with mature scar formation, whereas remote myocardium primarily contained fine fibers with limited assembly (Figure 3). This shift toward assembled fiber predominance suggested not only a difference in quantity but also in the maturity of fibrotic remodeling. To evaluate whether these compositional differences were accompanied by changes in organizational complexity, we performed fibrillar entropy mapping. Entropy analysis confirmed that peri-papillary regions exhibited markedly greater fibrillar disorder, with heterogeneous and disorganized collagen networks (red/yellow regions), while remote myocardium maintained uniformly low entropy indicative of a more ordered and stable fibrillar structure (Figure 4). Thus, peri-papillary remodeling was characterized by both fiber maturation and structural disarray, pointing to a unique regional fibrotic phenotype.

### 3.3. Endocardial vs. Myocardial Fibrosis Differ in Morphology

**Figure 5:**
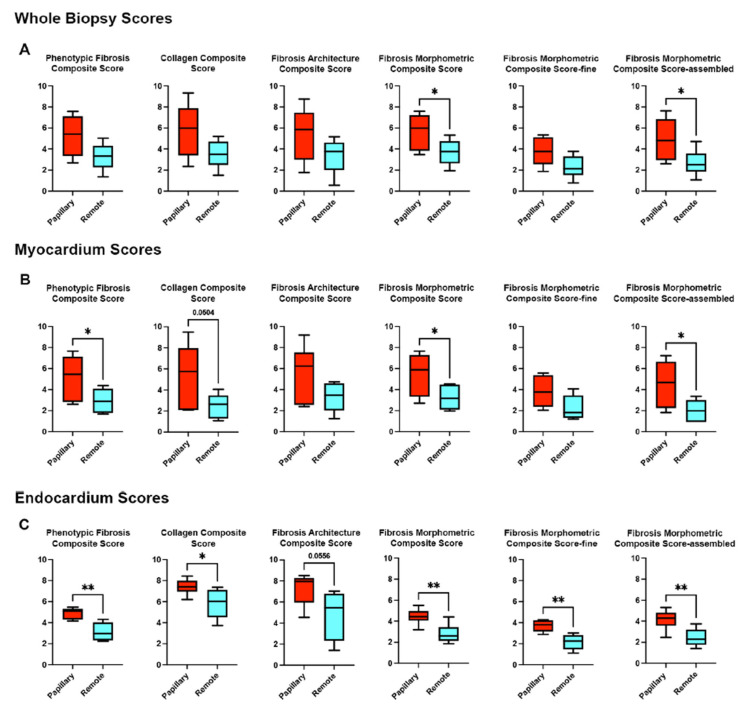
Quantitative fibrosis scoring across biopsy regions. Composite fibrosis scores derived from quantitative digital pathology analysis of biopsies from six MVP patients. (**A**) Whole biopsy scores demonstrate significantly higher fibrosis burden in peri-papillary versus remote regions, particularly in morphometric and assembled fiber metrics. (**B**) Myocardium-specific scores reveal higher phenotypic fibrosis, morphometric, and assembled fiber scores in papillary myocardium. (**C**) Endocardium-specific scores highlight striking differences, with papillary endocardium demonstrating significantly greater phenotypic fibrosis, collagen composite, and morphometric scores, including both fine and assembled fiber categories. * *p* < 0.05; ** *p* < 0.01.

Region-of-interest (ROI) segmentation allowed separate analysis of endocardial and myocardial components within the same biopsy sample (Figure S2). Integration of structural and architectural features across tissues yielded quantitative composite fibrosis scores, enabling comparison between peri-papillary and remote regions at the whole biopsy, myocardial, and endocardial levels (Figure 5). At the whole-biopsy level, peri-papillary regions consistently exhibited higher phenotypic fibrosis, collagen content, and morphometric scores, including assembled fiber measures. Layer-specific analyses revealed that myocardial remodeling was particularly pronounced adjacent to the papillary muscle, with higher phenotypic and assembled fiber scores, while endocardial regions showed the most dramatic differences, including increased collagen content and both fine and assembled fiber categories.

## 4. Discussion

Myocardial fibrosis has emerged as a critical pathophysiological feature in patients with mitral valve prolapse (MVP), particularly those at risk of sudden cardiac death [3,13,15,17,18]. Postmortem studies have consistently revealed inferobasal or mid-myocardial scarring in MVP patients with fatal arrhythmias, often in the absence of severe mitral regurgitation (MR) [8]. Similarly, intraoperative imaging and histopathology have demonstrated fibrosis in up to 86% of surgical MVP cases, though prior studies have largely relied on qualitative or semi-quantitative assessments. Cardiac MR imaging has expanded these observations by demonstrating late gadolinium enhancement and abnormal T1 mapping in MVP, with fibrosis correlating to arrhythmia risk, abnormal stain patterns, and adverse remodeling [5]. Despite >99% procedural success rates at high-volume mitral valve repair centers, approximately 20% of patients develop postoperative left ventricular (LV) dysfunction [26]. Together, these modalities underscore that myocardial fibrosis is both common clinically and consequentially in MVP, yet the timing, architecture, and potential reversibility of this remodeling remain incompletely understood.

In this study, we provide direct histologic and QIA–based evidence that mature, replacement-type myocardial fibrosis is already present at the time of surgical mitral valve repair. Using FibroNest digital pathology, we identified a significant increase in assembled collagen fibers, a hallmark of an organized scar, in the inferobasal myocardium adjacent to the papillary muscle, with minimal increase in fine collagen fibrils. This high-resolution morphometric approach advances prior histopathology by confirming MVP-associated fibrosis is not merely reactive or interstitial but represents structurally entrenched scar tissue. The regional distribution of this fibrosis aligns with CMR findings of inferobasal enhancement and strain abnormalities, reinforcing a mechanobiological model in which repetitive stress from leaflet prolapse and papillary tethering drives focal injury and scar formation.

These observations carry important clinical implications. First, they suggest that fibrotic remodeling in MVP begins earlier and progresses more extensively than previously recognized, raising concern that deferring intervention until overt mitral regurgitation or symptomatic progression may allow irreversible myocardial scarring. Second, the presence of mature fibrosis at surgery provides a plausible mechanism for postoperative LV dysfunction in 20% of patients, even after technically successful valve repair. In this context, our data complement prior imaging studies suggesting partial recovery of strain or function post-surgery, while clarifying that established scar is unlikely to regress, thereby reframing ongoing debates about the reversibility of MVP-associated fibrosis.

Our study demonstrates the power of digital pathology platforms such as FibroNest to move beyond descriptive pathology. Originally developed for liver fibrosis [21,22,25,27], this platform enables unbiased, reproducible, and high-dimensional assessment of collagen architecture, offering spatial and morphometric insights that are not achievable with conventional methods. While myocardial biopsies are not routinely used in clinical cardiology, this technology could be transformative in research settings, supporting preclinical antifibrotic drug development, validating noninvasive imaging surrogates, or stratifying patients based on fibrotic burden and remodeling patterns. Importantly, the detailed spatial and morphometric resolution of platforms like FibroNest allows for regional and compartment-specific insights, such as distinguishing endocardial vs. myocardial fibrosis, which may be critical in understanding arrhythmogenic substrates in MVP and other conditions.

In addition to mechanical stress, genetic predisposition plays a central role in MVP pathogenesis and myocardial remodeling. MVP can arise in both monogenic and polygenic forms, and it is observed in syndromic contexts as well as non-syndromic presentations. These observations suggest that germline genetic variation not only predisposes to leaflet prolapse but may also directly influence remodeling within the left ventricular myocardium. Importantly, our histopathological findings underscore that it is the regional localization of fibrosis, concentrated in inferobasal myocardium and papillary muscle insertion zones, that reveals the intersection of three drivers: genetic susceptibility, focal mechanical stress, and the cumulative passage of time. Together, these converging factors shape a disease process in which scarring represents the final common outcome.

Finally, our findings raise a forward-looking question: if MVP-associated fibrosis is already mature by the time symptoms or MR severity prompt surgical referral, should thresholds for intervention be reevaluated? There may exist a therapeutic window, prior to scar formation, during which valve repair could better preserve myocardial integrity, reduce arrhythmia risk, and optimize long-term outcomes. While this hypothesis requires validation in larger, longitudinal studies, our results provide a compelling rationale to revisit existing guidelines and trial designs for MVP management.

### Limitations

This study has limitations that should be recognized. First, the cohort size was small (n = 6) and derived from a single center, which limits generalizability. Although the findings are consistent across patients, they should be interpreted as hypothesis-generating and require validation in larger cohorts. Second, biopsy collection during the study period was necessarily limited to patients in whom intraoperative sampling was feasible, introducing potential selection bias. Third, long-term clinical follow-up, including post-operative imaging and arrhythmia outcomes, was incomplete, preventing correlation of histological findings with functional recovery or adverse events. Lastly, the Ph-FCS and other derived scores may be subject to overfitting, given the limited sample sizes and absence of independent validation cohorts. As such, their performance should be interpreted with caution. Due to the regional heterogeneity of myocardial tissue, sampling variability may limit the utility of the phenotypic scores presented in this study.

Despite these limitations, the findings provide meaningful insights and represent a valuable contribution to advancing digital pathology approaches for fibrosis assessment in cardiac tissue. Future studies integrating genetic profiling, imaging, and histopathology will be critical to fully delineate the mechanisms driving myocardial remodeling in MVP and to more rigorously define how these pathways might eventually inform the optimal timing of surgical intervention.

## 5. Conclusions

Our findings demonstrate that mature, regionalized myocardial fibrosis is prevalent in MVP patients at the time of surgical valve repair, particularly in regions subjected to high mechanical stress. The presence of assembled collagen fibers suggests that this fibrosis is persistent and likely contributes to postoperative LV dysfunction. These data underscore the importance of early detection and intervention in MVP and highlight the potential for digital pathology to reshape the way cardiac fibrosis is quantified, interpreted, and clinically integrated.

## Data Availability

All data will be made available following correspondence with the senior author.

## Supplementary Materials

The following supporting information can be downloaded at: https://www.mdpi.com/article/10.3390/cells14191536/s1, Figure S1: Quantitative Image Analysis Pipeline; Figure S2: Endocardial and Myocardial ROI Assignment; Table S1: Individual qFTs used for Composite Score Generation.
