# Supplementary figures and images for: Collagen Fiber Maturity and Architecture in MVP-Associated Fibrosis Quantified by Digital Pathology
Source: Cells. 2025 Sep 30;14(19):1536. doi: 10.3390/cells14191536 (PMC12523760; doi:10.3390/cells14191536)

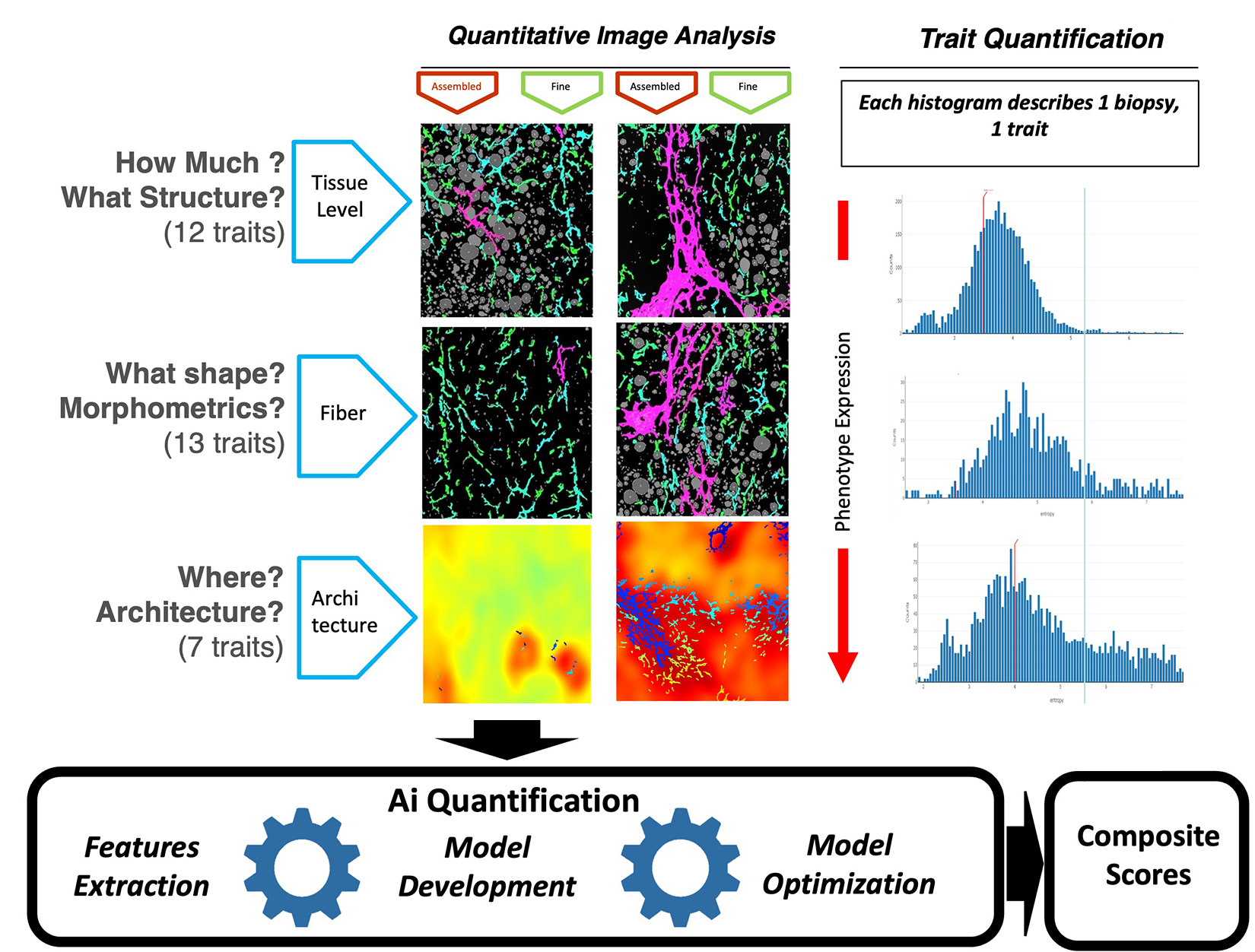

Supplement: Supplementary file 1 [file cells-14-01536-s001.zip › FINAL Figure S1.jpg]

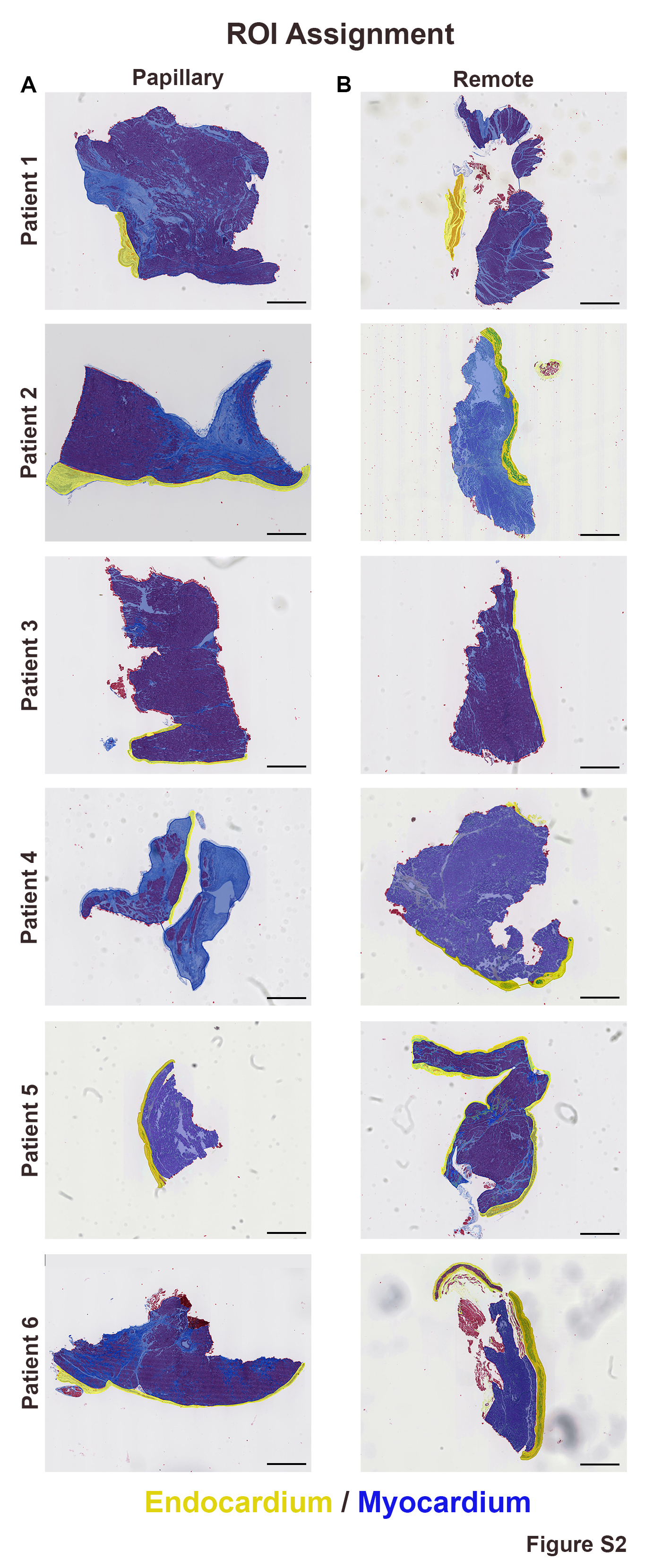

Supplement: Supplementary file 1 [file cells-14-01536-s001.zip › FINAL Figure S2.jpg]
